# Supplementary material for: An open-source framework for large-scale, flexible evaluation of biomedical text mining systems
Source: J Biomed Discov Collab. 2008 Jan 29;3:1. doi: 10.1186/1747-5333-3-1 (PMC2276192; doi:10.1186/1747-5333-3-1)
Supplement: Additional file 1 — Evaluation platform source code. The additional file contains the software evaluation framework discussed in this paper. The most current release can be downloaded from SourceForge.net [1]. See the accompanying README file for instructions on installation and use. Also included in the distribution is a collection of UIMA wrappers for some commonly used BioNLP tools and annotated corpora. [file 1747-5333-3-1-S1.zip › bionlp_uima_repository-1.0.1/docs/api/edu/uchsc/ccp/uima/ae/util/filter/OverlappingAnnotationResolver_AE.html]

OverlappingAnnotationResolver\_AE (Center for Computational Pharmacology Util/UIMA API)


|  |  |  |
| --- | --- | --- |
| **Overview** \| **Package** \| **Class** \| **Tree** \| **Deprecated** \| **Index** \| **Help** | |  |
| **PREV CLASS**   **NEXT CLASS** | **FRAMES**    **NO FRAMES**     **All Classes** |  |
| SUMMARY: NESTED \| FIELD \| CONSTR \| METHOD | DETAIL: FIELD \| CONSTR \| METHOD |  |


---


## edu.uchsc.ccp.uima.ae.util.filter Class OverlappingAnnotationResolver\_AE

```
java.lang.Object
  org.apache.uima.analysis_engine.annotator.Annotator_ImplBase
      org.apache.uima.analysis_engine.annotator.JTextAnnotator_ImplBase
          edu.uchsc.ccp.uima.ae.util.filter.OverlappingAnnotationResolver_AE
```

**All Implemented Interfaces:**: org.apache.uima.analysis\_engine.annotator.BaseAnnotator, org.apache.uima.analysis\_engine.annotator.JTextAnnotator

---

``` public class OverlappingAnnotationResolver_AE extends org.apache.uima.analysis_engine.annotator.JTextAnnotator_ImplBase ```

Default behavior is to keep the longest annotation for any that overlap. The user also has the option to keep the longest common substring among
all overlapping annotations.
  
  
Known bug in longest common substring:  
If you have three overlapping annotations  
11111111  
222222222222222222  
333333333333333333333333  
  
It is possible that the code will compare annotations 1 and 2 first, and result in a longest common substring of 22, and then miss completely the
longest common substring between 2 and 3.   
  
LONGEST COMMON SUBSTRING FEATURE DOES NOT CURRENTLY WORK

**Author:**
:   williamb

---

| **Field Summary** | |
| --- | --- |
| `protected  Set<Integer>` | `annotationSetsToIgnore` |
| `protected  String` | `annotationTypeOfInterest` |
| `protected  boolean` | `keepLongestCommonSubstring` |
| `String` | `PARAM_ANNOTATION_SETS_TO_IGNORE` |
| `String` | `PARAM_ANNOTATION_TYPE_OF_INTEREST`             The annotation type to resolve overlaps |
| `String` | `PARAM_KEEP_LONGEST_COMMON_SUBSTRING` |


**Constructor Summary**

`OverlappingAnnotationResolver_AE()`


| **Method Summary** | |
| --- | --- |
| `protected  boolean` | `checkForIgnoreBasedOnAnnotationSet(CCPTextAnnotation ccpTA)` |
| `void` | `initialize(org.apache.uima.analysis_engine.annotator.AnnotatorContext ac)` |
| `protected  void` | `keepLongestAnnotation(Map<Integer,CCPTextAnnotation> annotationIDMap, Map<Integer,Boolean> annotationIDRemovedMap, List<CCPTextAnnotation> annotationsToRemove, org.apache.uima.jcas.JCas jcas)`             keeps the longest annotation for a group of overlapping annotations |
| `protected  void` | `keepLongestCommonSubstring(Map<Integer,CCPTextAnnotation> annotationIDMap, Map<Integer,Boolean> annotationIDRemovedMap, List<CCPTextAnnotation> annotationsToRemove, org.apache.uima.jcas.JCas jcas)`             keeps the longest common substring for a group of overlapping annotations |
| `void` | `process(org.apache.uima.jcas.JCas jcas, org.apache.uima.analysis_engine.ResultSpecification arg1)`             Default behavior is to keep the longest of any overlapping annotation |

**Methods inherited from class org.apache.uima.analysis\_engine.annotator.Annotator\_ImplBase**

`destroy, finalize, getContext, getTypeSystem, reconfigure, typeSystemInit`

**Methods inherited from class java.lang.Object**

`clone, equals, getClass, hashCode, notify, notifyAll, toString, wait, wait, wait`

**Methods inherited from interface org.apache.uima.analysis\_engine.annotator.BaseAnnotator**

`destroy, reconfigure, typeSystemInit`

**Field Detail**

### PARAM\_ANNOTATION\_SETS\_TO\_IGNORE

```
public final String PARAM_ANNOTATION_SETS_TO_IGNORE
```

**See Also:**: Constant Field Values

---


### PARAM\_KEEP\_LONGEST\_COMMON\_SUBSTRING

```
public final String PARAM_KEEP_LONGEST_COMMON_SUBSTRING
```

**See Also:**: Constant Field Values

---


### PARAM\_ANNOTATION\_TYPE\_OF\_INTEREST

```
public final String PARAM_ANNOTATION_TYPE_OF_INTEREST
```

:   The annotation type to resolve overlaps

    **See Also:**: Constant Field Values

---


### annotationSetsToIgnore

```
protected Set<Integer> annotationSetsToIgnore
```

---


### annotationTypeOfInterest

```
protected String annotationTypeOfInterest
```

---


### keepLongestCommonSubstring

```
protected boolean keepLongestCommonSubstring
```


**Constructor Detail**

### OverlappingAnnotationResolver\_AE

```
public OverlappingAnnotationResolver_AE()
```


**Method Detail**

### initialize

```
public void initialize(org.apache.uima.analysis_engine.annotator.AnnotatorContext ac)
                throws org.apache.uima.analysis_engine.annotator.AnnotatorInitializationException,
                       org.apache.uima.analysis_engine.annotator.AnnotatorConfigurationException
```

:   **Specified by:**: `initialize` in interface `org.apache.uima.analysis_engine.annotator.BaseAnnotator` **Overrides:**: `initialize` in class `org.apache.uima.analysis_engine.annotator.Annotator_ImplBase`
:   **Throws:**: `org.apache.uima.analysis_engine.annotator.AnnotatorInitializationException`: `org.apache.uima.analysis_engine.annotator.AnnotatorConfigurationException`

---


### process

```
public void process(org.apache.uima.jcas.JCas jcas,
                    org.apache.uima.analysis_engine.ResultSpecification arg1)
             throws org.apache.uima.analysis_engine.annotator.AnnotatorProcessException
```

:   Default behavior is to keep the longest of any overlapping annotation

    :   **Throws:**: `org.apache.uima.analysis_engine.annotator.AnnotatorProcessException`

---


### keepLongestAnnotation

```
protected void keepLongestAnnotation(Map<Integer,CCPTextAnnotation> annotationIDMap,
                                     Map<Integer,Boolean> annotationIDRemovedMap,
                                     List<CCPTextAnnotation> annotationsToRemove,
                                     org.apache.uima.jcas.JCas jcas)
```

:   keeps the longest annotation for a group of overlapping annotations

    :   **Parameters:**: `annotationIDMap` -: `annotationIDRemovedMap` -: `annotationsToRemove` -: `jcas` -

---


### keepLongestCommonSubstring

```
protected void keepLongestCommonSubstring(Map<Integer,CCPTextAnnotation> annotationIDMap,
                                          Map<Integer,Boolean> annotationIDRemovedMap,
                                          List<CCPTextAnnotation> annotationsToRemove,
                                          org.apache.uima.jcas.JCas jcas)
```

:   keeps the longest common substring for a group of overlapping annotations

    :   **Parameters:**: `annotationIDMap` -: `annotationIDRemovedMap` -: `annotationsToRemove` -: `jcas` -

---


### checkForIgnoreBasedOnAnnotationSet

```
protected boolean checkForIgnoreBasedOnAnnotationSet(CCPTextAnnotation ccpTA)
```


---


|  |  |  |
| --- | --- | --- |
| **Overview** \| **Package** \| **Class** \| **Tree** \| **Deprecated** \| **Index** \| **Help** | |  |
| **PREV CLASS**   **NEXT CLASS** | **FRAMES**    **NO FRAMES**     **All Classes** |  |
| SUMMARY: NESTED \| FIELD \| CONSTR \| METHOD | DETAIL: FIELD \| CONSTR \| METHOD |  |


---
